# Comparing Single vs. Combined Cerebrospinal Fluid Parameters for Diagnosing Full-Term Neonatal Bacterial Meningitis

**DOI:** 10.3389/fneur.2019.00012

**Published:** 2019-01-23

**Authors:** Heyu Huang, Jintong Tan, Xiaohui Gong, Jing Li, Liping Wang, Min Xu, Xi Zhang, Yongjun Zhang, Lisu Huang

**Affiliations:** ^1^Pediatric Infectious Department, Xinhua Hospital Affiliated to Shanghai Jiao Tong University School of Medicine, Shanghai, China; ^2^Neonatal Department, Xinhua Hospital Affiliated to Shanghai Jiao Tong University School of Medicine, Shanghai, China; ^3^Neonatal Department, Children's Hospital of Shanghai Jiao Tong University, Shanghai, China; ^4^Neonatal Department, Shanghai Children's Medical Center Affiliated to Shanghai Jiao Tong University School of Medicine, Shanghai, China; ^5^Clinical Research Unit, Xinhua Hospital Affiliated to Shanghai Jiao Tong University School of Medicine, Shanghai, China

**Keywords:** bacterial meningitis, cerebrospinal fluid, term neonates, ambispective cohort study, combined CSF parameters

## Abstract

**Objectives:** To identify and compare the cerebrospinal fluid (CSF) parameters that predict the presence of neonatal bacterial meningitis using optimal cutoff values, and to derive and compare predictive profiles based on a combination of individual parameters for the same purpose.

**Study Design:** The retrospective component of the Shanghai Neonate Meningitis Cohort included all term neonates who underwent lumbar puncture between 2000 and 2017. Those with severe neurological diseases, histories of ventricular drainage, or traumatic lumbar punctures were excluded. Reference ranges were determined for non-bacterial meningitis neonates based on the 5th, 25th, 50th, 75th, and 95th CSF parameter quantiles, and their relationships with age were calculated using generalized additive models that tested for linear relationships. The optimal cutoff value for each measured CSF parameter was calculated using receiver operating characteristic analysis and by deriving the Youden's index. Parameters with good diagnostic efficacies were combined to produce predictive profiles using logistic regression. The diagnostic efficacies of the single parameters and profiles were compared in neonates with confirmed bacterial meningitis.

**Results:** White blood cells (WBCs) in CSF showed a higher diagnostic ability for neonatal bacterial meningitis than CSF protein, glucose, lactate dehydrogenase, or chloride. The sensitivity and specificity of the diagnostic cutoff value for WBCs (20 × 10^6^/L) were 95.1 and 98.7%, respectively. Profiles based on CSF parameter combinations improved the specificities slightly to 99.0–99.7%. However, employing predictive profiles did not improve sensitivities, which remained at 95.1–96.0%.

**Conclusions:** Profiles for predicting neonatal bacterial meningitis improve the sensitivity and specificity of diagnosis slightly, although not appreciably, compared to the single parameter of CSF WBC alone.

## Introduction

Bacterial meningitis more commonly occurs in the first month of life than at any other time (1). Although the mortality rate of neonatal bacterial meningitis has declined (2, 3), the morbidity rate remains relatively unchanged (4, 5). Almost half of the survivors remain at high risk of neurologic sequelae and lifelong impairment as a result of the infectious insult to developing brains (6). Early diagnosis is crucial for favorable prognosis, and delays in diagnosis and antibiotic treatment are independent risk factors for mortality (7).

Since the rate of bacterial meningitis-positive cerebrospinal fluid (CSF) cultures is quite low, diagnosing this condition still relies on deeper interpretation of CSF parameters. Some studies proposed that combined these parameters to build a predictive profile may improve the efficacy of diagnosing bacterial meningitis in neonates (8–10). However, pertinent studies have used small sample sizes and non-specific inclusion criteria (11, 12), which in turn skewed the interpretations of their results (13, 14). For example, most studies included infants, children, and sometimes even adolescent participants. It has been reported that the normal ranges for CSF parameters vary according to gestational age, chronological age, and birth weight (12, 15–18). This raises questions as to whether these diagnostic criteria are applicable to neonates and if these parameters are suitable as diagnostic indicators for newborns. Hence, we used data from our large multi-center cohort study to evaluate the diagnostic accuracies of CSF parameters individually or combined to produce different profiles. We also compared the efficacies of these single parameters and derived profiles in terms of diagnosing bacterial meningitis neonates.

## Methods

### Study Population

The Shanghai Neonate Meningitis Cohort is a multi-centered ambispective group that encompasses almost all term neonates who underwent lumbar puncture (LP) in Shanghai. The Cohort was derived from four tertiary class A pediatric hospitals that cover >95% of child meningitis admissions in Shanghai: Xinhua Hospital Affiliated to Shanghai Jiao Tong University School of Medicine, Shanghai Children's Medical Center Affiliated to Shanghai Jiao Tong University School of Medicine, Children's Hospital of Shanghai Jiao Tong University, and Children's Hospital of Fudan University. Our study only used retrospective data from the cohort and included all 2,802 neonates who underwent LP between January 2000 and December 2017. Approval for the study and data sharing with the coordinating institution was granted by the Institutional Review Board of each hospital (ethical approval No. XHEC-C-2017-084, clinical trial registration No. NCT03499652). The informed consent requirement was waived owing to the study's retrospective nature.

### Definitions and Classification Criteria

**Figure 1:**
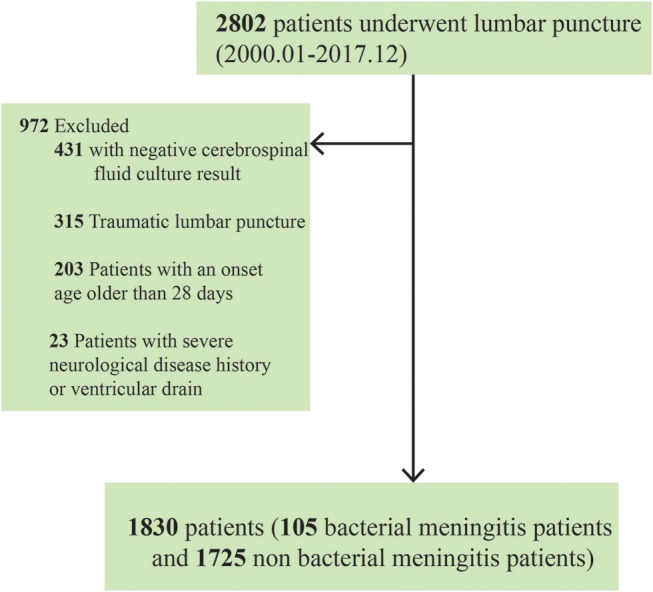
Flowchart of the participants' recruitment process.

Medical records were reviewed by neonatologists who extracted demographic data and information on clinical and laboratory findings. Neonates with positive CSF culture results were diagnosed with bacterial meningitis. However, we excluded neonates with positive cultures under any of the following circumstances: (1) the neonates experienced traumatic LP (i.e., >1,000 × 10^6^/L red blood cells in their CSF); (2) the neonates were >28 days at the onset of bacterial meningitis; and (3) a history of other severe neurological diseases or ventricular drainage was present (17, 19, 20). Finally, 1,830 neonates (105 with bacterial meningitis and 1,725 without) were included in our final retrospective analysis (Figure 1). All samples submitted for routine bacteriological culture at every participating hospital were handled according to local practice.

CSF parameters including white blood cell (WBC) counts as well as neutrophil, protein, glucose, lactate dehydrogenase (LDH), and chloride levels were obtained from the first LP after admission. Under 5% of the patients were missing data on WBCs, proteins, glucose, LDH, and chloride. Early-onset bacterial meningitis was defined as the onset of symptoms within the first 72 h of life.

### Statistical Analysis

Descriptive statistics were used to summarize the baseline characteristics; values are presented as the median as well as the 5th, 25th, 75th, and 95th quantiles. The differences in the clinical characteristics of the bacterial and non-bacterial meningitis groups were assessed using the χ^2^ test, Fisher's exact test, Kruskal-Wallis test, or independent *t*-test, as appropriate. The correlations between CSF parameters and age were calculated using generalized additive models for linear relationships.

We performed receiver operating characteristic (ROC) analyses with the data bootstrapped 500 times; the areas under the curve (AUCs) for CSF WBCs, protein, glucose, LDH, and chloride in neonates with and without bacterial meningitis were compared. To establish diagnostic profiles, we first considered five CSF parameters by using crude logistic regression models. All parameters significantly associated with bacterial meningitis in the crude models (i.e., *P* < 0.20) were selected as potential profile components. Finally, we developed four profile groups (Y1–Y4) each based on up to five parameters using the final multivariable logistic regression formula. Each single parameter was multiplied by its regression coefficients, and the sum of all these products as well as the constant term was deemed the final combined profile. We also calculated the Youden's index for different CSF parameter cutoff values, and applied the Delphi method to determine the threshold values for diagnosis (21). A panel of neonatologists, pediatric infectious diseases specialists, microbiologists, and clinical technicians was assembled as the consulting group. We compared the diagnostic performance of single and combined parameters by calculating their sensitivity, specificity, AUCs, and positive and negative predictive values with respect to bacterial meningitis in neonates.

All statistical analyses were performed using SPSS 20 (IBM Corp., Armonk, NY) and the R programming software. The graphs and figures were created using GraphPad Prism 5.0 (GraphPad Software, San Diego, CA) and SPSS 20.

## Results

### Demographic Characteristics and Clinical Symptoms

Pathogenic bacteria were identified from the CSF of 105 patients. Approximately half (49.5%) of the neonates with bacterial meningitis were born of local Shanghai residents; 3.2% of mothers received antibiotics before or during labor and approximately one-third (35.8%) of neonates were treated with antibiotics before admission. Twelve of the patients (11.4%) had early-onset disease. Neonates with bacterial meningitis were older than those without bacterial meningitis (13.8 ± 7.9 vs. 9.6 ± 8.9 days, *P* < 0.001), and their average stay in the hospital was much longer (32.9 ± 20.1 vs. 12.3 ± 9.1, *P* < 0.001). The top three reported clinical symptoms of neonates with bacterial meningitis were fever (84.8%), neurological symptoms (50.5%), and feeding difficulties (38.1%) (Table 1).

**Table 1:** Clinical characteristics of neonates with and without full-term bacterial meningitis.

	**Bacterial meningitis (*n* = 105)**	**Non-bacterial meningitis (*n* = 1725)**	***P*-value**
BASIC INFORMATION			
Male, n (%)	53 (50.5)	1045 (60.6)	0.040
Residence	47 (49.5)	961 (58.1)	0.097
Age (d), mean ± SD	13.8 ± 7.9	9.6 ± 8.9	<0.001
Birth weight (g), mean ± SD	3267 ± 499	3344 ± 554	0.172
Apgar < 7, *n* (%)	3 (5.8)	91 (5.3)	0.875
Age < 3 days, *n* (%)	12 (11.4)	679 (40.7)	<0.001
Cesarean delivery, *n* (%)	64 (68.1)	1446 (88.0)	<0.001
Multiple gestation times, *n* (%)	41 (39.4)	849 (49.3)	0.050
Multi-parity, *n* (%)	29 (27.9)	573 (33.3)	0.253
Maternal infection^a^, *n* (%)	13 (12.4)	282 (16.4)	0.278
Maternal peripartum antibiotic usage, *n* (%)	3 (3.2)	43 (2.6)	0.713
Neonatal antibiotic usage, *n* (%)	34 (35.8)	393 (23.8)	0.067
Referral, *n* (%)	42 (43.8)	518 (30.9)	0.071
Admission days (d), mean ± SD	32.9 ± 20.1	12.3 ± 9.1	<0.001
CLINICAL CHARACTERISTICS			
Fever, *n* (%)	89 (84.8)	846 (49.1)	<0.001
Neurological symptoms^b^, *n* (%)	53 (50.5)	386 (22.4)	<0.001
Feeding difficulties, *n* (%)	40 (38.1)	394 (22.8)	<0.001
Respiratory infections, *n* (%)	38 (36.2)	715 (41.4)	0.288
Mental status changes, *n* (%)	38 (36.2)	410 (23.8)	0.004
Jaundice, *n* (%)	24 (22.9)	600 (34.8)	0.159
Diarrhea, *n* (%)	11 (10.5)	166 (9.6)	0.774
Cyanosis, *n* (%)	6 (7.7)	147 (8.5)	0.795
Omphalitis, *n* (%)	7 (6.7)	74 (4.3)	0.252
Skin infection, *n* (%)	3 (2.9)	40 (2.3)	0.724
Urinary infection, *n* (%)	1 (1.0)	165 (9.6)	0.003

a *Maternal infection was diagnosed before or during labor according to medical files*.

b *Positive neurological symptoms were diagnosed as convulsion, dysmyotonia, irritability*.

*N, number; M, male; d, days; SD, standard deviation*.

### CSF Parameter Values as a Function of Bacterial Meningitis and of Age

**Figure 2:**
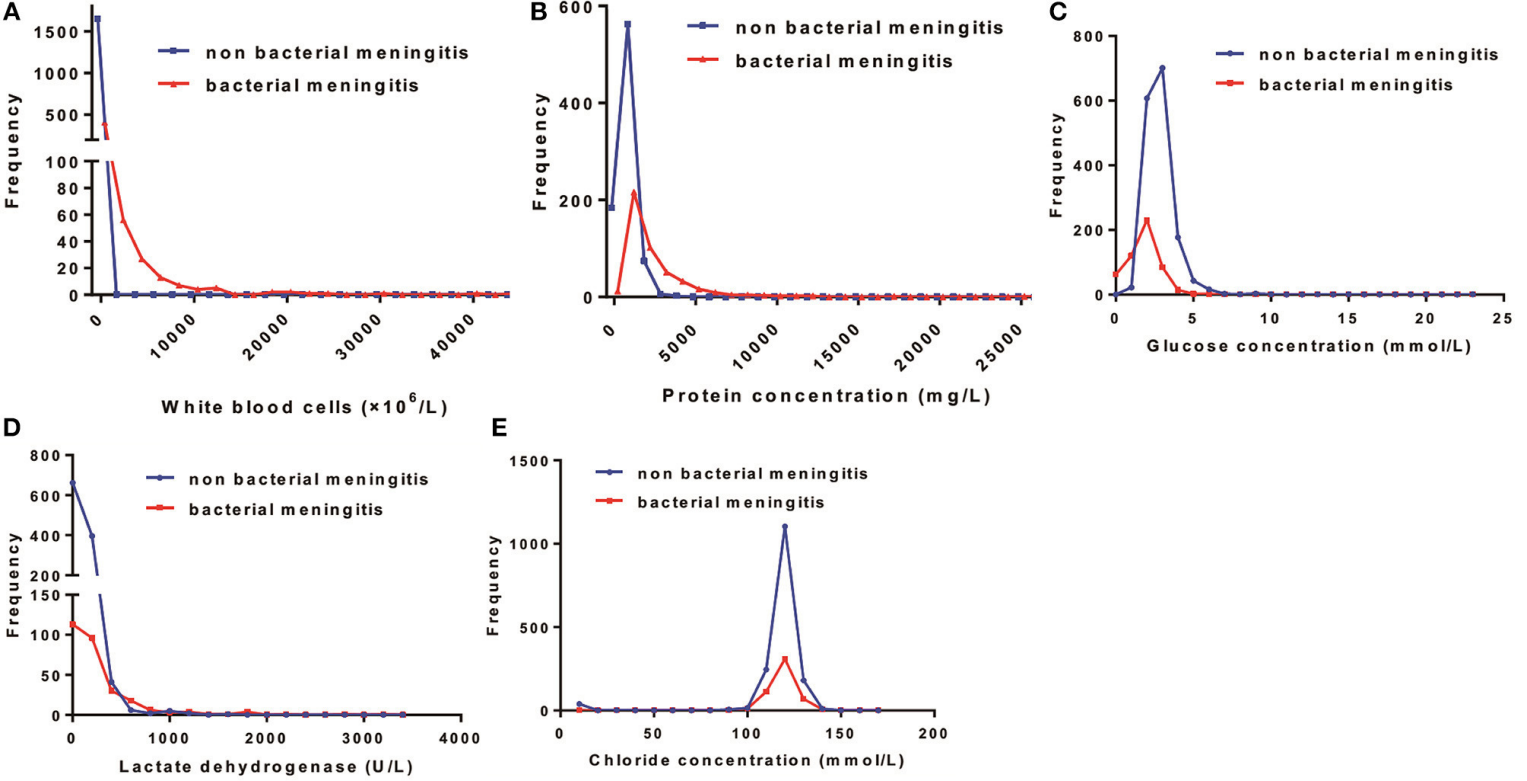
Distribution patterns of cerebrospinal fluid (CSF) parameters in the total bacterial and non-bacterial meningitis neonates. The distribution patterns of **(A)** white blood cells, **(B)** protein concentration, **(C)** glucose concentration, **(D)** lactate dehydrogenase, and **(E)** chloride concentration in neonates with and without bacterial meningitis.

The median WBC counts, protein levels, and LDH levels in neonates with bacterial meningitis were much higher than in those without this condition. CSF WBCs in neonates with bacterial meningitis ranged from 114 to 3,150 × 10^6^/L (Supplementary Table). In contrast, 95% of neonates without bacterial meningitis had <11 × 10^6^/L WBCs in CSF (Table 2 and Figure 2). CSF WBC counts barely overlapped between the two neonate groups.

**Table 2:** Cerebrospinal fluid parameters in non-bacterial meningitis neonates.

**Parameter**	**Quantile**				
	**5th**	**25th**	**50th**	**75th**	**95th**
White blood cells (10^6^/L)	0	0	2	5	11
Protein (mg/L)	325	555	747	1000	1536
Glucose (mmol/L)	2.0	2.0	3.0	3.0	4.0
Lactate dehydrogenase (U/L)	20	35	56	192	295
Chloride (mmol/L)	108	116	119	122	127

Generally, the median CSF protein and LDH levels in neonates with bacterial meningitis were higher than in neonates without, while the median CSF glucose concentration was lower (Supplementary Table). Protein, glucose, LDH, and chloride levels overlapped markedly between the two groups (Figure 2), implying a limited role for these factors in distinguishing between patients with vs. without bacterial meningitis. Protein concentrations >1,000 mg/L were found in 25% of neonates without bacterial meningitis and in >75% of those with the infection. The glucose concentrations in all neonates without bacterial meningitis were >2.0 mmol/L, while still one-quarter of neonates with the disease had glucose levels >2.0 mmol/L (Table 2).

In the non-bacterial meningitis group, neonates of younger chronological age had higher CSF WBC counts (*P* = 0.05) and protein concentration than older neonates, indicating that these values decreased with age during the neonatal period. In particular, protein levels were markedly higher in younger neonates (960 mg/L) than in older counterparts (580 mg/L; *P* < 0.001) (Supplementary Figure).

### The Diagnostic Efficacies of Single CSF Parameters and Their Combinations

**Figure 3:**
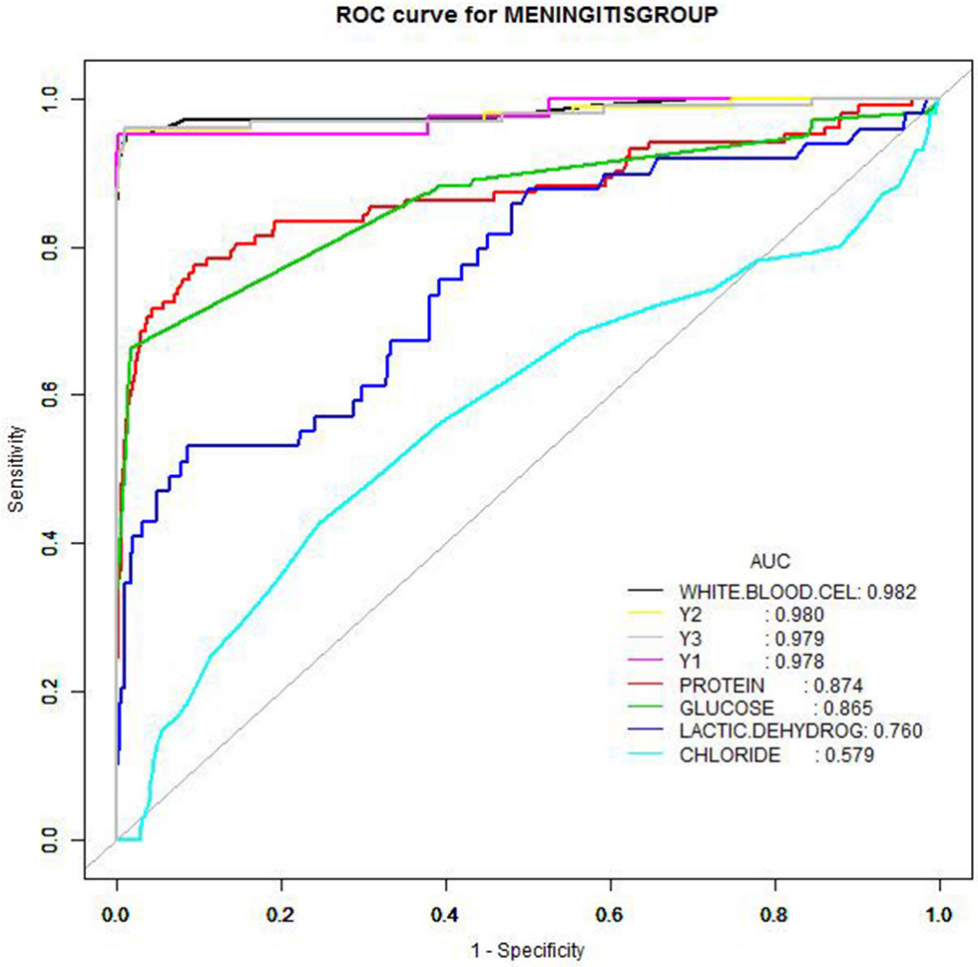
Receiver operating characteristic curves of cerebrospinal fluid parameters. Receiver operating characteristic curves of cerebrospinal fluid parameters (white blood cells, neutrophils, protein, glucose, lactate dehydrogenase, chloride) as well as parameter combinations to create diagnostic profiles, as a function of culture-proven bacterial meningitis patients.

The AUC values of CSF WBC counts (sensitivity 95.1%, specificity 98.7%) were remarkably better than for other parameters. Considering the practicality of acquiring WBC counts in the clinic, a threshold value of 20 × 10^6^/L was chosen for CSF WBCs using the Delphi method. The sensitivities and specificities of the CSF protein, glucose, and LDH values were lower (Table 3 and Figure 3). Protein and glucose concentrations exhibited good specificities (90.2 and 98.2%, respectively) but poor sensitivities (77.5 and 66.3%, respectively). The specificity of LDH was similar to that of protein, although its sensitivity was only 53.1%. Neither the specificity (75.4%) nor sensitivity (42.6%) of chloride concentration was acceptable (Table 3).

**Table 3:** Diagnostic efficacies of cerebrospinal fluid parameters estimated using receiver operating curves in bacterial meningitis neonates.

**CSF parameters**	**Cutoff values**	**AUC**	**95% CI**	**Specificity**	**Sensitivity**	**LLR+**	**LLR–**^**a**^
White blood cells (10^6^/L)	19.5	0.982	(0.964, 1.000)	0.987	0.951	74.72	0.05
Protein (mg/L)	1299.5	0.874	(0.825, 0.922)	0.906	0.775	8.21	0.25
Glucose (mmol/L)	1.95	0.865	(0.818, 0.913)	0.982	0.663	36.05	0.34
Lactate dehydrogenase (U/L)	271.5	0.760	(0.678, 0.837)	0.909	0.531	5.80	0.52
Chloride (mmol/L)	115.5	0.579	(0.512, 0.645)	0.754	0.426	1.73	0.76
Combined parameters Y1^**b**^	−1.7032	0.978	(0.947, 1.000)	0.997	0.951	336.41	0.05
Combined parameters Y2^**c**^	−2.8547	0.980	(0.959, 1.000)	0.990	0.958	97.75	0.04
Combined parameters Y3^**d**^	−2.8012	0.979	(0.956, 1.000)	0.991	0.960	101.14	0.04

a *Cutoff values were determined by calculating the Youden index*.

b *Y1 = −8.47839 + 0.19469 × white blood cells + 0.00190 × protein + 0.01667 × glucose – 0.00503 × lactate dehydrogenase*.

C *Y2 = −6.34939 + 0.14552 × white blood cells + 0.00069 × protein – 0.05915 × glucose*.

d *Y3 = −6.56709 + 0.14571 × white blood cells + 0.00071 × protein*.

*CSF, cerebrospinal fluid; AUC, area under the curve; CI, confidence interval; LLR+, positive likelihood ratio; LLR–, negative likelihood ratio*.

The CSF parameters were combined to produce four diagnostic profiles (Y1–Y4). While combining the protein, glucose, LDH, and/or chloride improved the sensitivity to 95.1–96.0% and the specificity to 99.1–99.7%, these changes were not appreciable.

## Discussion

In this large-scale multi-centered cohort, CSF WBC counts showed a high sensitivity and specificity in diagnosing bacterial meningitis. However, CSF protein, glucose, LDH, and chloride levels showed considerable overlap in patients with and without bacterial meningitis. Combining WBCs, protein, glucose, LDH, and chloride to create a diagnostic profile improved the specificity to some degree, but not sensitivity.

Most studies concerning the diagnostic thresholds of bacterial meningitis have largely focused on children, with certain proportions of neonates included in their analyses. Our study included only term neonates, and our results indicated that the most optimal diagnostic cutoff value is 20 × 10^6^/L for CSF WBCs. In some single center studies with small sample sizes, a WBC cutoff of 21 × 10^6^/L was considered the best upper limit (12–14, 20). The largest cohort published to date involves a multi-centered study that included infants delivered at ≥34 weeks of gestational age; a cutoff of 21 × 10^6^/L produced a sensitivity of 79% and a specificity of 81% (20). In our study, we found a much higher sensitivity and specificity (95.1 and 98.7%, respectively).

It was previously proposed that combining parameters like neutrophils percentage, protein level, and glucose concentration may improve the efficacy of diagnosis of neonatal bacterial meningitis (22–24). Oostenbrink et al. derived an equation that incorporated the CSF protein and glucose values to predict which children suspected of having bacterial meningitis would require empirical antibiotic treatment (9). Using CSF parameters and clinical symptoms, Nigrovic et al. developed a bacterial meningitis score to assist clinicians identify children with CSF pleocytosis who were at very low risk of bacterial meningitis (10). Furthermore, other researchers even found that a combination of serum procalcitonin and CSF LDH achieved the highest predictive power for bacterial meningitis in adolescents and adults, with a sensitivity and specificity exceeding 99% each (24).

The permeability of the blood-brain barrier increases in patients with bacterial meningitis (25); neutrophils quickly migrate to the infection site across the blood-brain barrier and are believed to play important roles in attacking pathogens, thus leading to tissue damage (26–28). In our study, neutrophils were not detected in neonates with CSF WBCs < 10 × 10^6^/L; approximately 95% of neonates without bacterial meningitis also had no neutrophils detected. Therefore, we did not consider this parameter because of the amount of missing data.

We found that protein, glucose, and LDH showed high specificities in diagnosing bacterial meningitis; hence, we attempted to derive diagnostic profiles using these parameters using logistic regression. When the Y1 profile score (including WBCs, protein, glucose, and LDH) was higher than −1.7032, there was 99.7% certainty that the neonate had bacterial meningitis; however, the sensitivity of this method was barely different from that of WBC alone. Protein concentrations are relatively constant in children and adolescents owing to their compact blood-brain barriers. When meningitis occurs, the disruption in the blood-brain barrier leads to increased CSF protein concentrations (27); thus, protein has always been used as a specific indicator of bacterial meningitis in these pediatric patients. In our study, CSF protein concentrations during the neonatal period decreased significantly with chronologic age to almost half of that on the first day. This might influence the efficacy of this parameter in diagnosing neonatal bacterial meningitis. Although we found that normal CSF WBCs values also markedly change with age (23), CSF WBCs only narrowly decreased with chronologic age.

Glucose is a frequently used indicator when diagnosing bacterial meningitis in adults, but compared to WBC, CSF glucose concentrations are more inconsistent in neonates (12, 23). As the sensitivity of CSF glucose concentration was low while the specificity was relatively high, we concluded that glucose concentration is not a stable parameter and could be influenced by the administration of intravenous glucose when neonates are hospitalized for LP. Furthermore, LDH was previously found to be reliable for distinguishing bacterial meningitis from the viral sort (29); however, the sensitivity and specificity of LDH in our study were not acceptable. This discrepancy with previous studies may be because our study only included neonates.

There were some limitations to our study. First, it was retrospective in nature, leading to inevitable bias when collecting clinical data from medical documents. To reduce recall bias, all medical files were proofread using the Delphi method. Second, we only included patients who underwent LP; hence, those with mild cases may have been missed. Although the patients were all supposed to receive follow-up for at least 6 months, this was rarely implemented; besides, including patients with mild bacterial meningitis would have only increased the sensitivities and specificities of the diagnostic cutoff values.

In conclusion, we found that the WBC count was a useful parameter for diagnosing bacterial meningitis in full-term neonates, with the infection likely to be present in neonates with WBCs above the cutoff of 20 × 10^6^/L. Combining the diagnostic parameters of WBCs, protein, glucose, LDH, and chloride boosted the specificity to 99.7%, but did not change the sensitivity.

## Author Contributions

The manuscript was written by HH, JT, and XG. Clinical data were collected by HH, LW, and MX. Clinical files were proofread by JT, JL, and XG. LH and XZ were in charge of data analysis and YZ was responsible for the coordination of this clinical research.

### Conflict of Interest Statement

The authors declare that the research was conducted in the absence of any commercial or financial relationships that could be construed as a potential conflict of interest.
