# Supplementary material for: Comparing Single vs. Combined Cerebrospinal Fluid Parameters for Diagnosing Full-Term Neonatal Bacterial Meningitis
Source: Front Neurol. 2019 Jan 23;10:12. doi: 10.3389/fneur.2019.00012 (PMC6351467; doi:10.3389/fneur.2019.00012)
Supplement: Supplementary file 1 [file Data_Sheet_1.docx]

**Supplementary Table: Characteristics of cerebrospinal fluid (CSF) parameters in neonates with and without bacterial meningitis**

| Cerebrospinal fluid parameters | Bacterial meningitis  Median (Q1, Q3) | Non-bacterial meningitis  Median (Q1, Q3) |
| --- | --- | --- |
| White blood cells (10^6^/L) | 820.0 (114.0, 3150.0) | 2.0 (0.0, 5.0) |
| Protein (mg/L) | 2343.5 (1374.8, 3980.0) | 747.0 (555.2, 999.8) |
| Glucose (mmol/L) | 1.0 (0.2, 2.0) | 3.0 (2.0, 3.0) |
| Lactic dehydrogenase (U/L) | 276.0 (104.0, 513.0) | 56.0 (35.0, 196.5) |
| Chloride (mmol/L) | 117.0 (113.0, 122.0) | 119.0 (116.0, 122.0) |

**Supplementary Figure: The relationship between cerebrospinal fluid protein concentration and age in neonates without bacterial meningitis**
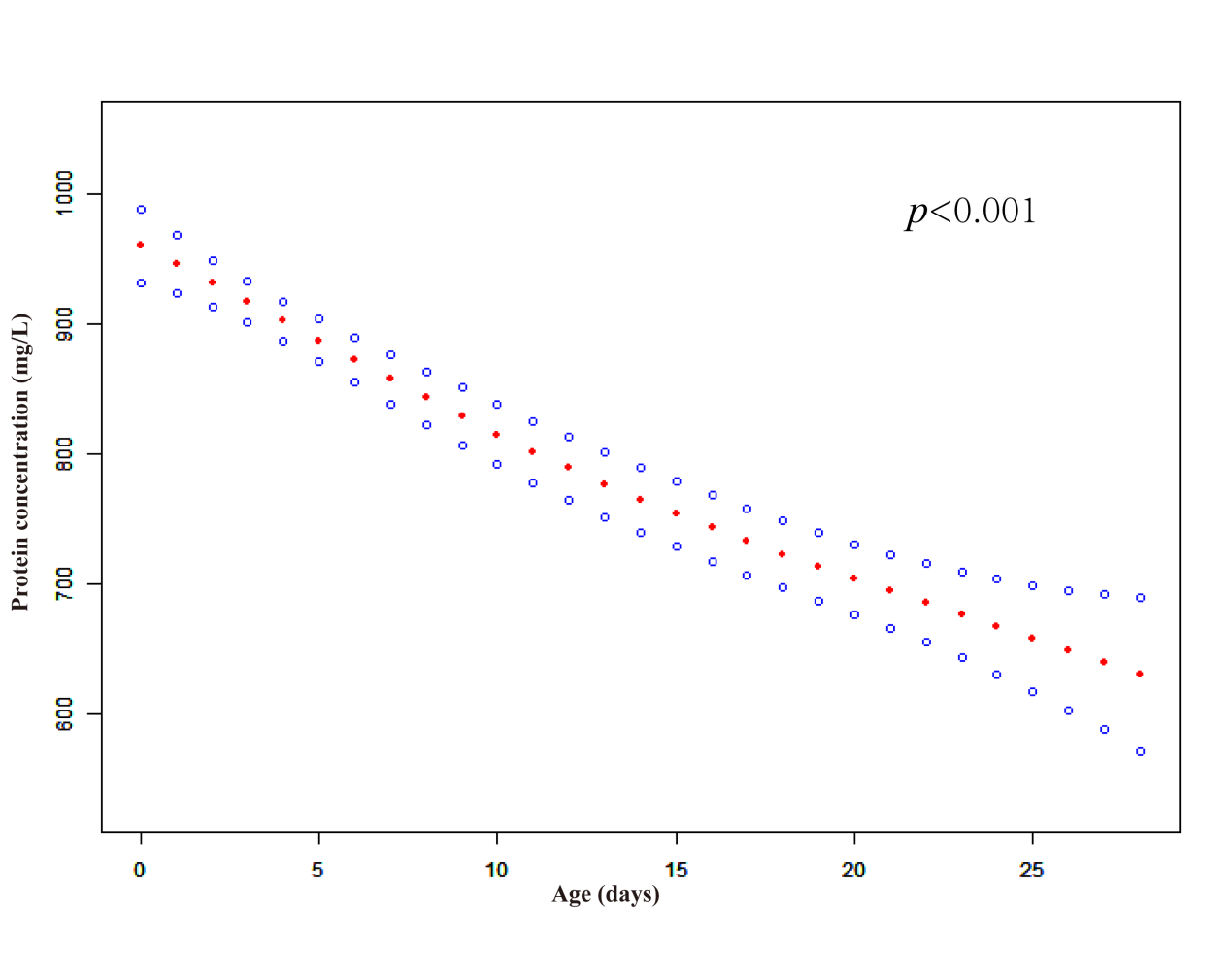


P value was calculated by generalize additive models to test for liner relationships between protein and age. This graph demonstrates the correlation between age and protein concentration derived from 1725 term non-bacterial meningitis neonates. From the curves, we can see protein concentration decreased as age increased in the neonatal period. Red dots in the graph stand for the predictive values of CSF protein concentration, blue dots stand for 95% confidential intervals of predictive values.
